# Advanced Biofuels Based on Fischer–Tropsch Synthesis for Applications in Gasoline Engines

**DOI:** 10.3390/ma14113134

**Published:** 2021-06-07

**Authors:** Jiří Hájek, Vladimír Hönig, Michal Obergruber, Jan Jenčík, Aleš Vráblík, Radek Černý, Martin Pšenička, Tomáš Herink

**Affiliations:** 1 Department of Chemistry, Faculty of Agrobiology, Food and Natural Resources, Czech University of Life Sciences Prague, Kamýcká 129, 165 00 Prague, Czech Republic; jirihajek@af.czu.cz (J.H.); obergruber@af.czu.cz (M.O.); jencikj@af.czu.cz (J.J.); 2 ORLEN UniCRE a.s., 436 01 Litvínov, Czech Republic; ales.vrablik@orlenunicre.cz (A.V.); radek.cerny@orlenunicre.cz (R.Č.); martin.psenicka@orlenunicre.cz (M.P.); tomas.herink@unipetrol.cz (T.H.)

**Keywords:** Fischer–Tropsch synthesis, biofuel, biogasoline, bionaphtha, biocomponent, alternative fuels, standardization, waste materials

## Abstract

The aim of the article is to determine the properties of fuel mixtures of Fischer–Tropsch naphtha fraction with traditional gasoline (petrol) to be able to integrate the production of advanced alternative fuel based on Fischer–Tropsch synthesis into existing fuel markets. The density, octane number, vapor pressure, cloud point, water content, sulphur content, refractive index, ASTM color, heat of combustion, and fuel composition were measured using the gas chromatography method PIONA. It was found that fuel properties of Fischer–Tropsch naphtha fraction is not much comparable to conventional gasoline (petrol) due to the high n-alkane content. This research work recommends the creation of a low-percentage mixture of 3 vol.% of FT naphtha fraction with traditional gasoline to minimize negative effects—similar to the current legislative limit of 5 vol.% of bioethanol in E5 gasoline. FT naphtha fraction as a biocomponent does not contain sulphur or polyaromatic hydrocarbons nor benzene. Waste materials can be processed by FT synthesis. Fischer–Tropsch synthesis can be considered a universal fuel—the naphtha fraction cut can be declared as a biocomponent for gasoline fuel without any further necessary catalytic upgrading.

## 1. Introduction

Replacing fossil fuels with biofuels and alternative fuels is currently a much-discussed topic regarding the energy future of the European Union and the Czech Republic. The European Union is one of the main players in the fight to reduce greenhouse gas emissions into the atmosphere. From the point of view of the long-term sustainability of transport, the two biggest problems are the consumption of fossil fuels and the related production of carbon dioxide emitted into the atmosphere. The tool for reducing greenhouse gas emissions is the use of renewable energy sources in energy and industry. The strategy in the field of biofuels is legislatively supported by the revised Directive 2018/2001 on Renewable Energy Sources (RED (I)) [1] and Directive 2009/28/EC on the promotion of the use of energy from renewable sources (RED (II)) [2].

The Directive sets criteria for the sustainability and savings of greenhouse gas emissions for biofuels, bioliquids and biomass fuels. The directive supports the development of renewable energy sources over the next decade with a binding EU-wide target of at least 32% for renewable energy by 2030, to be achieved jointly by the Member States. To this end, the Directive contains a number of sectoral measures to promote the further use of energy from renewable sources in the electricity, heating, cooling and transport sectors, with the general aim of contributing to reducing greenhouse gas emissions, improving energy security, and strengthening Europe’s leadership in technologies and industry in terms of renewable energy and job creation and growth [3,4].

Each Member State shall ensure, from 1 January 2021, at least 70% savings in greenhouse gas emissions from the use of liquid and gaseous fuels from renewable sources of non-biological origin used in the transport sector. Furthermore, from 1 January 2021, the share of energy from renewable sources may not be lower than the basic share of energy from renewable sources in the given year. It is further stipulated that, within the 14% minimum share of final energy consumption in transport in each Member State, the share of advanced biofuels and biogas produced from raw materials (expressed as a share of final energy consumption in the transport sector) must be at least 0.2% in 2022, in 2025 at least 1%, and in 2030 at least 3.5%. These binding national targets are in line with the target of at least 32% of the share of energy from renewable sources in gross final energy consumption in 2030 in the European Union. Furthermore, a standardized formula for the calculation of greenhouse gas emission savings for fuels produced from biomass, including their given percentages, is introduced [1].

In the transport sector, improving the efficiency of combustion in engines and a significant increase in the use of biofuels and alternative propulsion are also the key to reducing greenhouse gas emissions. Biofuels have become an integral part of everyday life in modern society. Bioethanol and fatty acid methyl esters are a common part of commercially available blends of gasoline and diesel fuels. Pressure on replacing fossil fuels with alternative fuels and vehicles with alternative propulsions are constantly growing. Despite significant problems associated with high production costs, technical and logistical problems, and a negative impact on food prices for first-generation biofuels, today’s society is moving towards so-called advanced biofuels, the raw material base of which is agricultural non-food production and waste biomass [5].

Second-generation biofuels include ethanol and butanol [6,7] and BtL fuel produced by thermo-chemical processing into liquid synthetic fuel [8,9]. Such fuels are produced from the lignocellulosic forest biomass, including harvesting residues, agricultural waste (straw, hay, maize, rapeseed and other residues), energy plants (pterosaurs, sorghum, sorrel, etc.), as well as biological waste from households, used frying oil, waste animal fat and municipal waste [6]. Another widespread method is chemical recycling of waste using pyrolysis by producing pyrolysis oil. Sources for this process can be waste tyres [10], plastic waste [11], municipal solid waste [12] or lignocellulosic biomass [13,14]. Last but not least, in this category is also hydrogen produced from renewable energy sources [15].

According to the LCA analysis, advanced biofuels show a significant positive difference in the balance of CO_2_ production during their life cycle. Biofuels produced from these raw materials include bioethanol from lignocellulosic biomass, biomethanol or gasoline as a product of the catalytic conversion of synthesis gas, biobutanol and biodiesel produced by hydrogenation or transesterification of non-food raw materials or used cooking oils [16]. From the point of view of circular economics, however, the production of synthetic fuels formed after pyrolysis of waste plastics into pyrolysis oil or conversion of synthesis gas to Fischer–Tropsch synthesis products seems to be the most promising [17].

Depending on the reaction conditions, FT synthesis can be distinguished into low-temperature and high-temperature synthesis. Low-temperature FT synthesis (LTFT) is operated in the range of 200–250 °C. Both cobalt and iron catalysts are suitable for this application. Whereas, cobalt ones are more commonly used for lower temperatures of the temperature range. The products are mainly high molecular weight alkanes and linear waxes. For this synthesis, a tubular reactor with a solid bed of catalyst or a reactor with catalyst in suspension are most commonly used, which are usually more efficient [18].

High-temperature synthesis (HTFT) is characterized by operating temperatures around 320–350 °C using an iron-based catalyst. The product is essentially only low molecular weight alkenes in the form of gas and gasoline fractions at reaction conditions, thus producing an air-tight system without a liquid phase. The entire process is carried out in fluidized bed reactors and circulating catalyst reactors [18].

FT synthesis is a catalytic polymerization process occurring on the surface of a catalyst, which uses hydrocarbon monomers formed by hydrogenation of adsorbed CO to produce long-chain hydrocarbons with a wide range of applications. The reactions involve the adsorption and dissociation of reagents (CO and H_2_) specifically on the catalyst surface and the formation of chain initiators that will lead to chain growth and termination and subsequent desorption of the final products.

Since Fischer–Tropsch synthesis catalysts are mostly used in powder form, reactions with these materials imply nanoeffects. Such phenomena occur with nanomaterials, e.g., catalysts made totally or partially with nanostructured materials. Many sectors, for example, polymeric composite materials, are carrying out a lot of scientific and technological works and there are also plans for the wide range of projects being the nanomaterials in use. It has caught tremendous attention and interest in the promotion of nanostructured coatings. All this is because of the unique properties that are at hand, offering the possibilities of multifunctionality, reduction of thickness, and a great spectrum of applications related to technology.

However, recent works on nanoparticles showcase the potential risks of nanoparticle aerosol releases and allow a more balanced benefit/risk analysis [19]. For example, many studies highlight nanoparticle emissions due to coatings, paints [20], tiles [21]. Cases of nanoparticle exposure in the field of occupational hygiene by coating workplaces have been reported [22]. These exposures can occur also when using powders [23,24,25,26].

## 2. Materials and Methods

To determine the effect of FT-naphtha distillation fraction on winter gasoline, mixtures with working names were selected:

- 100 vol.% pure fossil gasoline (BA100).
- 3 vol.% FT-naphtha distillation fraction and 97 vol.% fossil gasoline (FT3).
- 5 vol.% FT-naphtha distillation fraction and 95 vol.% fossil gasoline (FT7).
- 10 vol.% FT-naphtha distillation fraction and 90 vol.% fossil gasoline (FT15).
- 25 vol.% FT-naphtha distillation fraction and 75 vol.% fossil gasoline (FT30).
- 50 vol.% FT-naphtha distillation fraction and 50 vol.% fossil gasoline (FT50).
- 70 vol.% FT-naphtha distillation fraction and 30 vol.% fossil gasoline (FT70).
- 100 vol.% pure FT-naphtha distillation fraction (FT100).

Pure fossil gasoline was produced by Unipetrol RPA and it is fully compliant with standard EN 228+A1 for winter class F/F1 without ethanol as a biocomponent. Winter gasoline was chosen because of possible year-long operability. In winter there can be a problem in the formation of fuel mixtures, a problem with low vapour pressure and with volatility. Mixture for the summer season has a vapor pressure margin.

The FT-naphtha distillation fraction by mass (BP 30–180 °C) was produced in an atmospheric/vacuum distillation column in UniCRE laboratories from FT products created within the COMSYN project [4].

Distillation of FT products was performed on laboratory distillation apparatus PILODIST. The PILODIST 105 distillation apparatus is a system with 70 theoretical plates and a Sulzer EX column packing. Atmospheric distillation and vacuum distillation of crude oil, petroleum products and materials of a similar nature can be performed on this unit. Atmospherically, the unit can be distilled up to 200 °C. subsequently, a certain degree of vacuum must be included in the process. The entire distillation system is connected to the control software DCD4001.

To identify the fuel properties of the mixtures, the physiochemical properties of FT-naphtha fraction were determined. In the evaluation of fuel density, octane number, colour, vapor pressure, cloud point, water content, PIONA content, refractive index, ASTM colour, and distillation curves were measured. These fuel properties were compared for fuels containing volumetric amounts of different FT-naphtha fraction. All measurements were conducted according to the valid standards. A list of them is given in Table 1.

Parameters were always measured three times and results represent the average value from three measurements with the expanded uncertainty with 95% confidence interval. The expanded uncertainty *U* of the measurand was obtained by multiplying the combined standard uncertainty *u*(*y*) by a coverage factor *k*, which gives the best estimate of the value attributable to the measurand. The value of the coverage factor k was chosen to meet the probability of coverage of about 95%, which for a normal distribution corresponds to the factor *k* = 2.

## 3. Results

### 3.1. Fuel Parameters

The most important property of fuels for spark ignition engines is the ability of the fuel to withstand the spontaneous/uncontrolled onset of oxidation reactions in the conditions of working circulation in the cylinder. This applies in particular to the phase of the progressive burning of the focus of ignition of the directions to the not yet ignited mixture [27].

**Figure 1:**
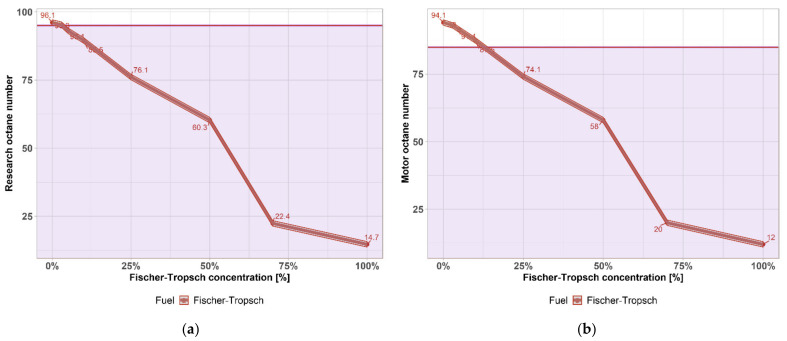
(**a**) Research octane number of FT–gasoline mixtures; (**b**) Motor octane number of FT–gasoline mixtures.

The octane number is the main quality parameter of gasoline and expresses how resistant fuel is to knock. If the fuel does not have sufficient anti-knock properties, detonation combustion will occur, which can lead to engine destruction. The octane number is characterized as the percentage of isooctane (2,2,4-trimethylpentane, octane number 100) and n-heptane (octane number 0) in the mixture, which has the same detonation resistance as the test fuel [28]. Two different engine operating modes were used to determine the octane number—the research method and the engine method (Figure 1a). The minimum values according to EN 228 for motor gasoline are shown in Figure 1b for both methods by a purple horizontal line.

As can be seen in Figure 1, the octane number of blends of gasoline with FT naphtha is greatly influenced by the octane numbers of the individual components. The lower octane number of FT naphtha compared to gasoline limits the use of the high-percentage FT-gasoline mixture to only the low percentage, as the high-percentage fuel was more prone to knocking and lower efficiency. The mixture already exceeded the lower limit of the standard for the tested mixtures by 5 vol.% FT naphtha in the research method and 15 vol.% FT naphtha in the engine method. This fact could lead to a requirement to adjust the composition of the gasoline pool for certain refinery configurations, in particular to reduce the addition of olefins and more pressure to mix isoalkane fractions with low fuel sensitivity (i.e., the difference in research octane number).

From a motor point of view, however, the reduced octane number can also be solved at higher speeds—less load, or for example throttling of the intake air, i.e., a smaller throttle angle (less “gas”). In general, reducing the compression ratio by 1 reduces the octane requirement of a gasoline engine by about 10–15 octane units, but this cannot be generalized, as even two different engines with the same compression ratio and cylinder capacity can differ significantly in their octane requirement.

**Figure 2:**
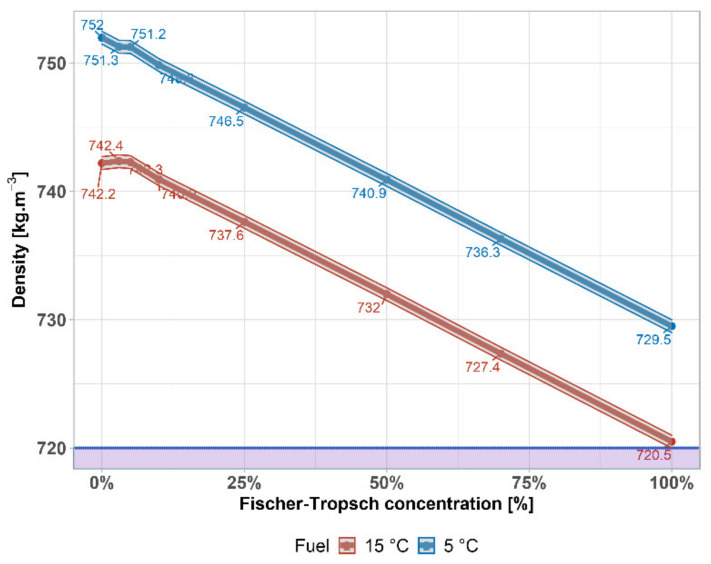
Density of FT–gasoline mixtures.

The density of gasoline is important, especially for commercial reasons, to be able to convert volumes to weights (weight), but it represents a certain relation to heat of combustion of the fuel. Figure 2 shows the decrease in density with the addition of FT naphtha. However, even at higher concentrations of FT naphtha (even at 100%), the fuel is within the EN 228 standard and because of it, the effect on engine power will not be significant. The minimum value according to EN 228 for density is shown by a purple horizontal line.

**Figure 3:**
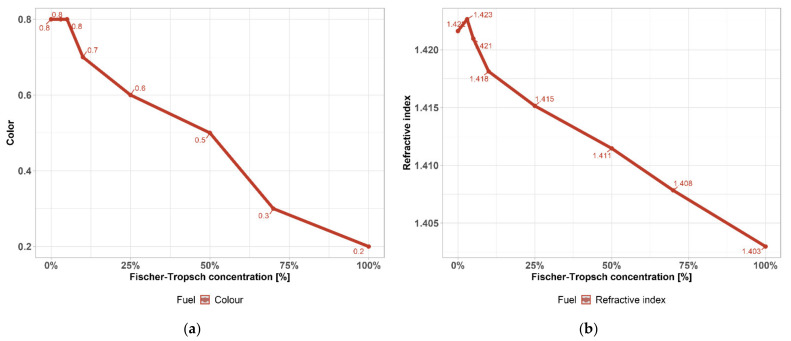
(**a**) Colour of FT–gasoline mixtures; (**b**) refractive index of FT–gasoline mixtures.

ASTM colour (Figure 3a) is a scale from 0.5 (lightest) to 8 (darkest), used for manufacture as fuel for automobile and marine engines, households, industry and the market. Determination of the colour of petroleum products is used mainly for manufacturing control purposes and is an important quality characteristic since colour is readily observed by the user of the product. In some cases, the colour may serve as an indication of the degree of refinement of the material. When the colour range of a particular product is known, a variation outside the established range may indicate possible contamination with another product. However, colour is not always a reliable guide to product quality and should not be used indiscriminately in product specifications. There are basic analyses for qualitative verification of petroleum intermediates of the product, colour indicates according to possible presence of heteroatom such as S, N and metals and according to refractive index change of hydrocarbon composition, paraffins aromatics, simple double bonds have different refraction, i.e., according to nature of HC chain. As the FT-to-naphtha ratio increases, which is pure, those values change to cleaner ones.

Measuring the refractive index of light (Figure 3b) as a way of indirect measuring the properties of liquids is a very efficient and relatively accurate method. It makes it possible to analyse practically all liquid materials, such as oils, antifreeze mixtures, brake or hydraulic fluids, leachates, paints, thinners, etc. It immediately displays specific investigated quantities. It is simply sufficient to determine the conversion coefficient/characteristic of the dependence of the change in the refractive index of the liquid on the given quantity.

### 3.2. Volatility Characteristics

**Figure 4:**
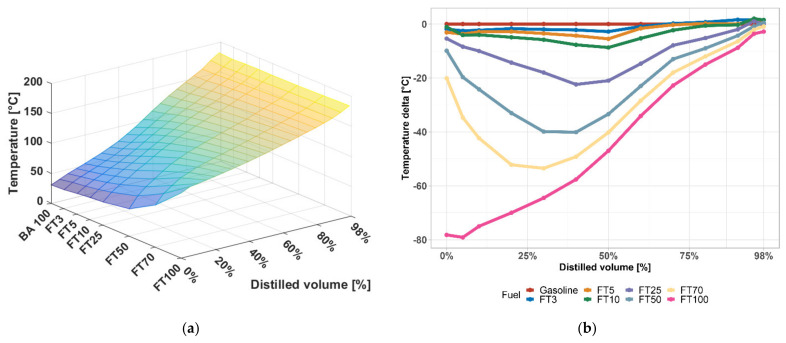
(**a**) Distillation curve of FT–gasoline mixtures; (**b**) changes in temperature distillation of gasoline containing FT naphtha.

The distillation curve of gasoline blends is another dominant test necessary to understand the properties of fuel. The beginning of distillation, which characterizes the boiling points of the lightest hydrocarbons, is especially important for the evaluation. As can be seen in Figure 4, FT naphtha increases the value of the onset of distillation, which on the one hand reduces fuel losses by evaporation during storage and bottling, but this increased value already suggests that a reduction in saturated vapor pressure can be expected.

Furthermore, it is necessary to monitor the value of 10% of the distilled amount of fuel. This expresses the ability of the fuel to generate a sufficient proportion of vapours even in the cold intake manifold so that the mixture in the cylinder is ignited by a spark on the spark plug in the range of 70–90 °C. A temperature below 80 °C is stated to be satisfactory; with existing gasoline it is usually 65–70 °C, which is also fulfilled by high-percentage mixtures of FT naphtha–gasoline [29].

A 50 vol.% of the distilled volume is referred to as the so-called middle or “core fraction”. At the 50% point it depends on the speed of engine warm-up, i.e., the speed at which its traction and power increase (starting the vehicle) after starting a cold engine. This value further affects the acceleration mode, i.e., the life of the vehicle. Importantly, even the high-percentage FT naphtha–gasoline mixtures did not exceed a temperature above 140 °C during the distillation curve for 50 vol.%, as the engine would then react noticeably slowly to the pedal depress [29].

The maximum temperature to which 95 vol.% of the fuel may be distilled, less often 97% of the fuel with boiling points higher than 200 °C, characterizes the end of the distillation curve. These hydrocarbons condense on the cylinder walls during combustion in the engine, where they dissolve the engine oil layer. However, the value of this point (95 vol.%) corresponds to 100% gasoline and the effect on the dilution of the engine oil with added FT diesel does not change. Dilution of engine oil is otherwise dangerous due to the low “viscosity reserve” of modern engine oils. Portions with a boiling point above 200 °C would not evaporate even in a hot engine, they remain in the form of droplets which only partially burn, a part is ejected into the cylinder wall by a vortex in the combustion chamber and subsequently dissolves in the motor oil layer to reduce the oil’s viscosity [30].

**Figure 5:**
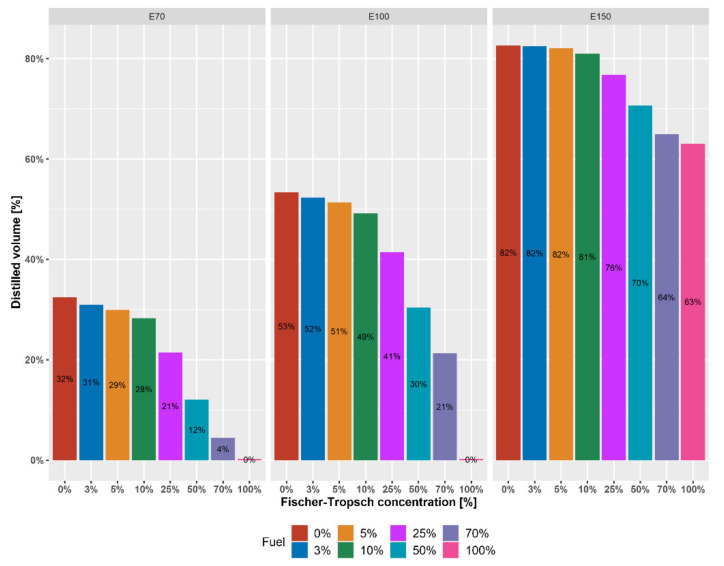
Influence of FT–naphtha on E70, E100 and E150 of FT–gasoline mixtures.

Changes in the distillation curve of gasoline with FT–naphtha are also associated with changes in other parameters. Figure 5 shows the differences in the amount of evaporated amount of standard values (EN 228 for gasoline) E70 at 70 °C, E100 at 100 °C and E150 at 150 °C depending on the amount of FT naphtha added.

Regarding the boiling points of individual hydrocarbons in mixture, the decrease of all three monitored values is noticeable. However, it is most pronounced for the E70 and E100 with the addition of FT naphtha. At the lower limit of the EN 228 standard for motor gasoline for parameter E70 (min. 20% vol.) is a mixture of 25% FT naphtha and higher. The same applies to E100, where the same mixture of 25% FT oil is below the standard (min. 46% vol.)

The reduction in the tendency is evident from the E150 value corresponding to the flattening of the distillation curve at 100% FT naphtha visible in Figure 4. Below the lower limit of the EN 228 standard for E100 (min. 75% vol.) is up to 50% FT naphtha.

The vapor pressure according to standard is the maximum pressure developed by the vapours of a given volume of sample in four times the volume of air in the prescribed apparatus under the test conditions.

**Figure 6:**
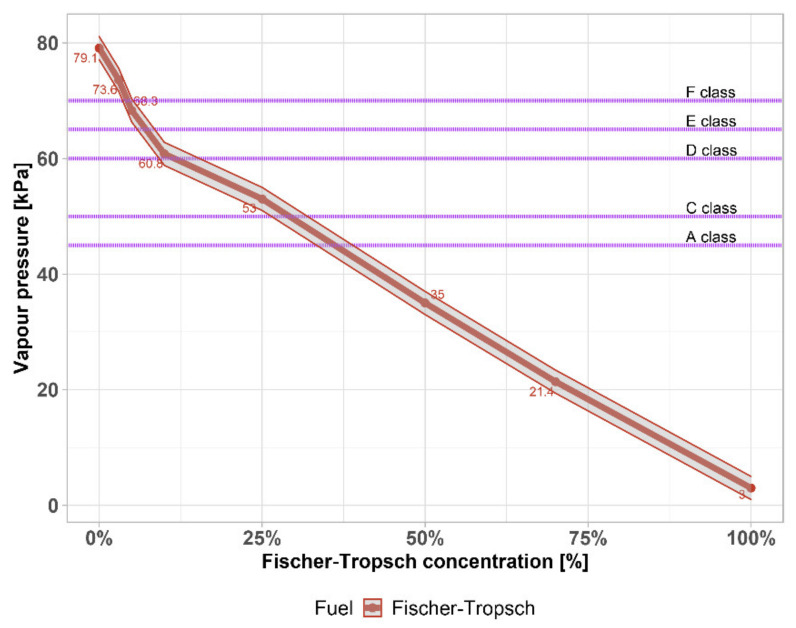
Vapour pressure of FT–gasoline mixtures.

**Figure 7:**
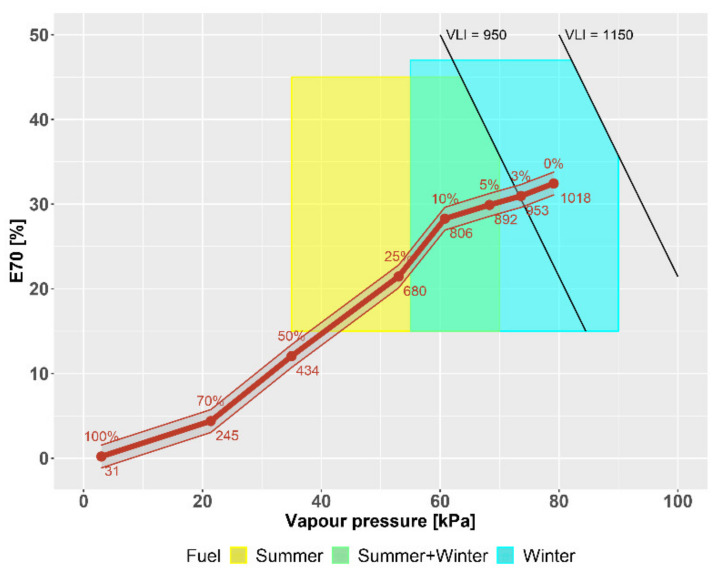
Vapour Lock Index (VLI) of FT–naphtha mixtures.

Figure 5 shows the effect of FT naphtha on the distillation process and Figure 6 on the vapor pressure of gasoline. These parameters are reflected in a decrease in the volatility index (Figure 7). For the potential use of high-percentage mixtures of FT naphtha–gasoline, there would be a problem with startability, especially at low temperatures, and it is necessary to use appropriate additives or create mixtures with gasoline with a higher reserve of vapor pressure.

The higher the pressure, the easier it is for the engine to start and the better it starts, which is of course associated with higher storage losses. In contrast to the usual addition of bioethanol to gasoline, there is no so-called commingling (mixing) effect, which is manifested by the formation of an azeotrope with a minimum boiling point, at which the pressure of the newly formed mixture increases between about 5–10%. Even for this, refineries must be prepared and take into account the reserve for fuel commonly referred to as E5 (EN 228–5% bioethanol in gasoline). However, if bioethanol were replaced by FT naphtha, this effect does not occur and, conversely, it is appropriate to choose gasolines with a higher vapor pressure (for example, winter marked in Figure 6 as F class). The lower vapor pressure limits for each type of gasoline are indicated in Figure 6 by a description of each class.

The Vapor Lock Index (VLI) in Figure 7, together with the amount of gasoline evaporated at 70 °C (distillation test E70) and vapor pressure, determines the amount of volatiles in the FT naphtha/gasoline mixture. The VLI assessment is important for easy engine starting, especially in winter, but during storage and pumping they evaporate, leaking into the air and causing gasoline losses. The VLI thus also determines the suitability of gasoline for the winter and summer periods. The volatility index is calculated from the vapor pressure in kPa and the vaporized amount of gasoline at 70 °C (E70) in % by volume. Changes in parameter E70 of gasoline with added FT naphtha as well as changes in vapor pressure result in a decrease in the volatility index. Due to the very low vapor pressure of pure FT naphtha, it is important to know the temperature dependence of gasoline mixtures with FT naphtha, since a certain minimum volatility of gasoline with a minimum vapor pressure of 5 kPa is required for a cold engine start [31,32].

This is the lower limit of the starting ability. This must be taken into account especially in winter, as the vapor pressure of the blended fuel is directly proportional to the temperature and the resulting usability in winter and summer can be seen in Figure 7. For high-percentage mixtures, it would be necessary to increase this parameter by adding a hydrocarbon component volatility. The reason is to meet the requirement for the minimum volatility of the fuel necessary for starting ability at lower temperatures. Thus, low-boiling components of gasoline, C4–C5 hydrocarbons, come into consideration. The use of these hydrocarbons in commercial gasolines is limited precisely because of the high vapor pressure, which may also be suitable for improving the final balance of the refinery.

### 3.3. Composition Analysis

**Figure 8:**
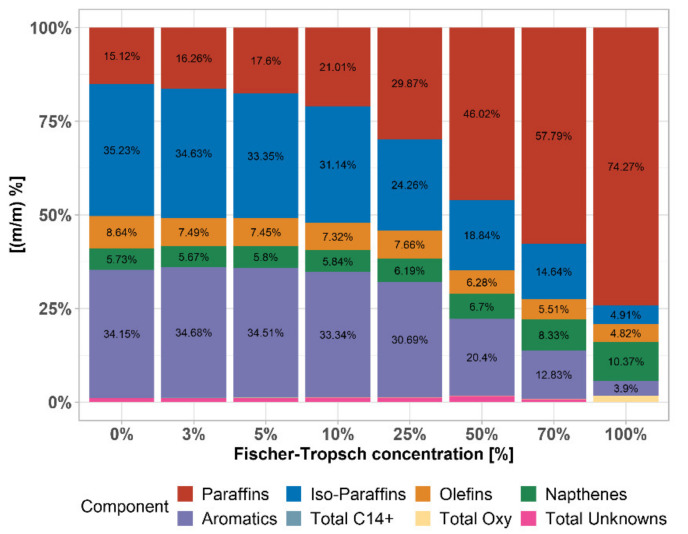
PIONA composition of FT–gasoline mixtures.

In Figure 8 we can see the exact composition of the main representatives of hydrocarbon compounds. For its determination analytical method PIONA was used. PIONA is an analysis used to determine the content of alkanes, iso-alkanes, olefins, naphthenes, and aromatics in gasoline fractions, as determined by multidimensional gas chromatography. As can be seen in the Figure 8, with the addition of the predominantly n-alkane FT naphtha fraction to fossil gasoline, which is predominantly a mixture of iso-alkanes and aromatics, the proportion of hydrocarbon groups gradually changes and thus the fuel properties of each mixture are changed.

## 4. Discussion

In the article were measured fuel properties, volatility characteristics and composition analysis was carried out, including octane number, density, colour, refractive index, distillation characteristics, vapour pressure, vapour lock index and PIONA composition. This measurement provides a full-scale analysis of the fuel properties so that an informed decision can be made as to the circumstances under which the blended fuel can be used. Fischer–Tropsch fuel was produced in a state-of-the-art atmospheric/vacuum distillation column in UniCRE laboratories within the COMSYN project [4].

It was found that octane number is greatly influenced by FT–naphtha and in the case of RON, standard EN 228 is fulfilled only for 3% mixture. Drop of octane number can be solved by adding an octane booster, but this possibility will be examined in a future article. For now, it was measured that 3% mixture can be used in any gasoline engine without a problem.

Density of FT–gasoline mixtures comply with EN 228 in whole interval. A slight drop around 20 kg·m^−3^ is caused by a gradual increase in the concentration of paraffins compared to iso-paraffins. This information was given by full PIONA analysis, providing the weight composition of mixture. It was found that FT–naphtha contains 74% paraffins against 15% in pure gasoline. On the other hand, FT–naphtha contains only 5% of iso-paraffins against 35% in pure gasoline. The hydrocarbon products in FT reaction follow the Anderson–Schulz–Flory (ASF) distribution. Because of this, FT synthesis is well set up for the production of paraffinic diesel [33,34,35].

Volatility characteristics give the information about the behaviour in an engine, cold start engine’s capability, operation stability, engine wear, etc. According to the results, high-percentage FT–naphtha would have problems with these properties since T70 and T100 are significantly low. The distillation curve is flatter than in gasoline which would not provide smooth burning in the engine.

The result of this work proves the possibility to add up to 3 vol.% FT naphtha fraction into traditional gasoline. Although this amount is low, it is justified at present by the small expansion and application of advanced alternative fuels of the second-generation gasoline fraction in EU countries, despite the benefits of double-counted emission savings according to RED II. It still dominates the obsolete first-generation application [36]. Low-percentage mixing is also supported by the legislative fact supported by the standardized parameter in EN 228 + A1. This standard limitation addition of ethanol as a biocomponent to a maximum of 5 vol.%. This result therefore makes the resulting added 3 vol.% FT a decrease in gasoline in the fuel, which can be done to include in the sale without conflict with current legislation. Registration is in the process of production of conventional gasoline and therefore has an ambitious increase by always reducing CO_2_ consumption without the cost of upgrading to high-percentage biofuel with the possible need for changes in legislation in the field of fuel sales.

## 5. Conclusions

This research work recommends the creation of a low-percentage mixture of 3 vol.% of FT naphtha fraction with traditional gasoline to minimize negative effects—similar to the current legislative limit of 5 vol.% of bioethanol in gasoline E5. Fischer–Tropsch FT naphtha fraction as a biocomponent does not contain sulphur or polyaromatic hydrocarbons nor benzene. Waste materials can be processed by FT synthesis.

Fischer–Tropsch synthesis can be considered a universal fuel-the naphtha fraction cut can be declared as a biocomponent for gasoline fuel without any further necessary catalytic upgrading.

Fischer–Tropsch is a promising concept for biorefineries preparing motor fuels from plant material or waste without any need for oil. It should be noted that Fisher–Tropsch synthesis fuels have the highest sustainability criteria when using waste materials and represent a long-term usable energy source.

## Figures and Tables

**Table 1:** Standards for the evaluation of the physicochemical properties.

Property	Standard
Diesel fuel	BS EN 590:2013 + A1:2017
Density	ISO 12185:1996
Distillation	ISO 3405:2011
Octane number	ISO 5164:2014
Refractive index	ISO 5661:1983
Colour	ASTM D1500
Vapour pressure	EN 13016-1
Multidimensional gas chromatography	ISO 22854:2016

## Data Availability

Data is contained within the article.
